# A Mobile-Based Deep Learning Model for Cassava Disease Diagnosis

**DOI:** 10.3389/fpls.2019.00272

**Published:** 2019-03-20

**Authors:** Amanda Ramcharan, Peter McCloskey, Kelsee Baranowski, Neema Mbilinyi, Latifa Mrisho, Mathias Ndalahwa, James Legg, David P. Hughes

**Affiliations:** ^1^Department of Entomology, College of Agricultural Sciences, Penn State University, State College, PA, United States; ^2^International Institute for Tropical Agriculture, Dar el Salaam, Tanzania; ^3^Department of Biology, Eberly College of Sciences, Penn State University, State College, PA, United States; ^4^Center for Infectious Disease Dynamics, Huck Institutes of Life Sciences, Penn State University, State College, PA, United States

**Keywords:** cassava disease detection, deep learning, convolutional neural networks, mobile plant disease diagnostics, object detection

## Abstract

Convolutional neural network (CNN) models have the potential to improve plant disease phenotyping where the standard approach is visual diagnostics requiring specialized training. In scenarios where a CNN is deployed on mobile devices, models are presented with new challenges due to lighting and orientation. It is essential for model assessment to be conducted in real world conditions if such models are to be reliably integrated with computer vision products for plant disease phenotyping. We train a CNN object detection model to identify foliar symptoms of diseases in cassava (*Manihot esculenta* Crantz). We then deploy the model in a mobile app and test its performance on mobile images and video of 720 diseased leaflets in an agricultural field in Tanzania. Within each disease category we test two levels of severity of symptoms-mild and pronounced, to assess the model performance for early detection of symptoms. In both severities we see a decrease in performance for real world images and video as measured with the F-1 score. The F-1 score dropped by 32% for pronounced symptoms in real world images (the closest data to the training data) due to a decrease in model recall. If the potential of mobile CNN models are to be realized our data suggest it is crucial to consider tuning recall in order to achieve the desired performance in real world settings. In addition, the varied performance related to different input data (image or video) is an important consideration for design in real world applications.

## 1. Introduction

Conventional plant disease diagnosis by human experts is inherently subjective and limited to regions that can support the required human infrastructure (Bock et al., 2010). Computer vision algorithms show promise to transform this field with the landmark result of a deep convolutional neural network (CNN) winning the Imagenet competition to classify over 1 million images from 1,000 categories, almost halving the error rates of its competition (LeCun et al., 2015). This success brought about a revolution in computer vision with CNN models dominating the approach for a variety of classification and detection tasks. As CNN models become the standard computer vision model to be deployed in real-time vision applications, assessing and reporting whether the results of their performance translates from research datasets to real time scenarios is crucial. Results of different CNN architectures are usually reported on standard large scale computer vision datasets of a million and more static images (He et al., 2016, 2017; Szegedy et al., 2016; Howard et al., 2017). Domain specific datasets like medical imagery or plant diseases, where transfer learning is often applied to CNN models, comprise smaller datasets as expert labeled images are more challenging to acquire (Masood and Ali Al-Jumaily, 2013; Ramcharan et al., 2017). In a recent assessment for a skin lesion classification task, researchers reported the performance of the deep learning model matched at least 21 dermatologists tested across three critical diagnostic tasks (Esteva et al., 2017). This study was done on a labeled dataset of 129,450 clinical images and the researchers concluded that the technology could be deployable on a mobile device but further evaluation in real-world settings is needed. Similar promising results have been shown in studies of plant disease classification (Mohanty et al., 2016; Johannes et al., 2017; Ramcharan et al., 2017) and health care (Miotto et al., 2017). Deploying on mobile devices would also be beneficial in democratizing access to algorithms while maintaining user privacy by running inference offline.

Despite the ubiquity of smartphones there are few examples of CNN models deployed on these phones categorizing visual scenes in the real world where performance is affected by input data type and compounded by wide extremes in lighting as is normal in outdoor settings. Clear examples of computer vision in real world settings such as autonomous vehicles (cars and drones) leverage multiple sensors in both the visible and non-visible spectrum (Floreano and Wood, 2015; Janai et al., 2017). If mobile CNN models are to achieve their promise it is important to recognize the constraint of a single sensor (i.e., camera) and test the performance of a CNN on mobile devices in conditions they are intended to be used in.

Here, we investigate plant disease diagnostics on a mobile device. We deploy and test the performance of a CNN object detection model for real-time plant disease diagnosis in an agricultural field. Within each disease category we test two levels of severity of symptoms - mild and pronounced, to assess the model performance for early detection of symptoms. We report precision, recall, F-1 score, and accuracy for mobile images and video to assess how the CNN performs in a real world app with different types of input data.

## 2. Methods

**Figure 1:**
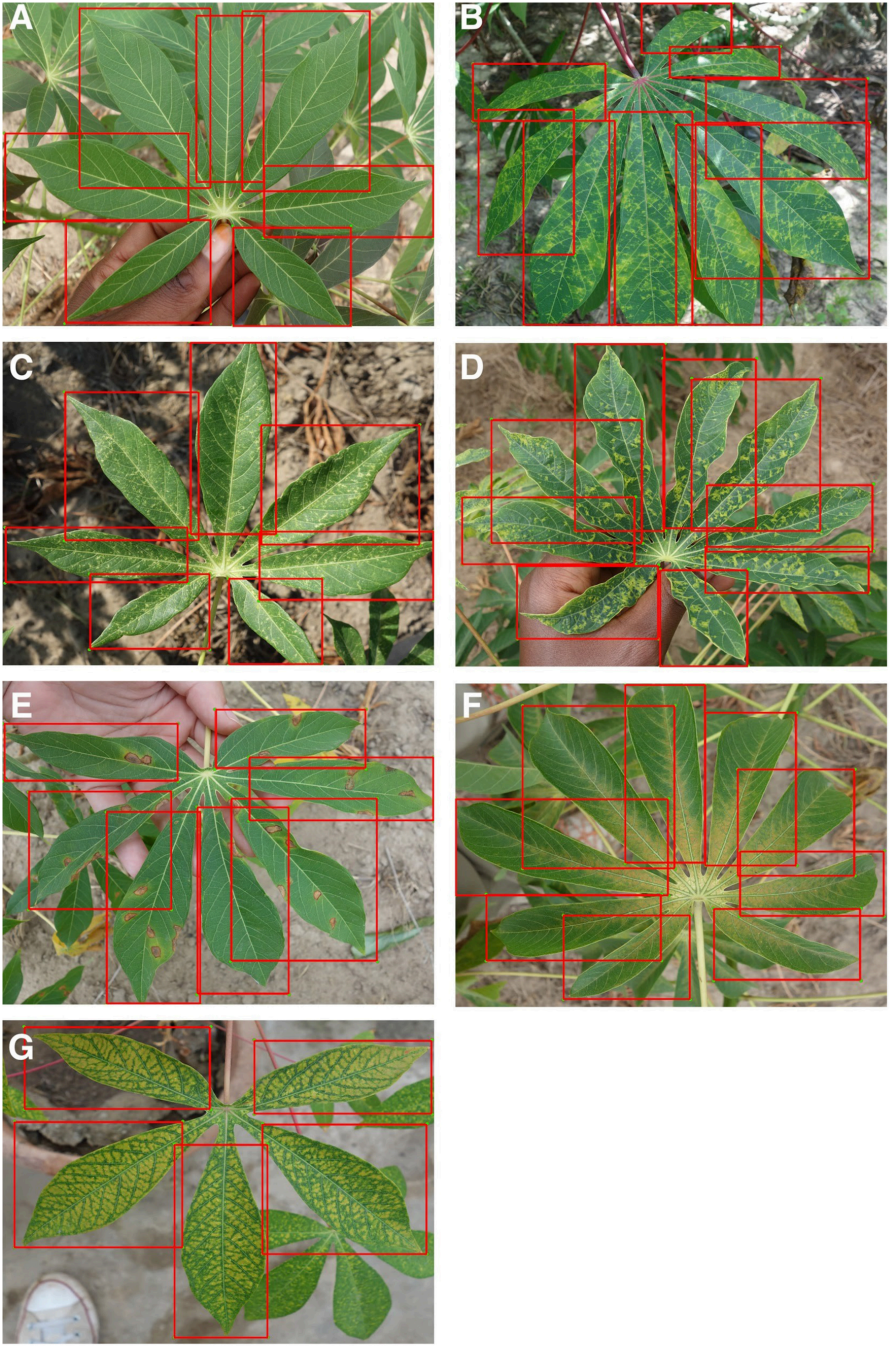
Examples of training images from 7 classes with leaflet annotations. Classes are **(A)** Healthy, **(B)** Brown streak disease, **(C)** Mosaic disease, **(D)** Green mite damage, **(E)** Red mite damage, **(F)** Brown leaf spot and **(G)** Nutrient Deficiency.

We use the Tensorflow platform to deploy a smartphone CNN object detection model designed to identify foliar symptoms of three diseases, two types of pest damage, and nutrient deficiency (or lack thereof) in cassava (*Manihot esculenta* Crantz). We utilize the Single Shot Multibox (SSD) model with the MobileNet detector and classifier pre-trained on the COCO dataset (Common Objects in Context) of 1.5 million images (80 object categories). For simplicity, we refer to the CNN object detector model as the mobile CNN model. We employ transfer learning to fine tune the model parameters to our dataset which comprised 2,415 cassava leaf images for pronounced symptoms of each class. The cassava leaf dataset was built with images taken in experimental fields of the International Institute of Tropical Agriculture (IITA), in Bagamoyo District, Tanzania. Complete details of this dataset were previously reported in Ramcharan et al. (2017). In addition to the 6 image classes implemented in Ramcharan et al. (2017), an additional nutrient deficiency class of 336 images was included in this work and examples of all image classes are shown in Figure 1.

For this study, three cassava disease experts reviewed images and agreed on classifications. Images were then annotated at Penn State University. Initially three different annotation styles were tested to identify class objects: (1) whole leaflet - object bounding boxes are drawn around leaflets with visible symptoms and boxes contain the leaf edges, (2) within leaflet—object bounding boxes are drawn around visible symptoms, inside of leaflets only, and do not contain leaf edges, and (3) combined inside and whole leaflet—annotation style (1) and (2) are combined with the same class labels for whole leaflet and within leaflet bounding boxes. Based on training results to 500 epochs (see Supplementary Material) on two 16Gb NVIDIA V100 GPUs the whole leaflet annotation style recorded the lowest overall loss and was selected to test on a mobile device in the field.

**Figure 2:**
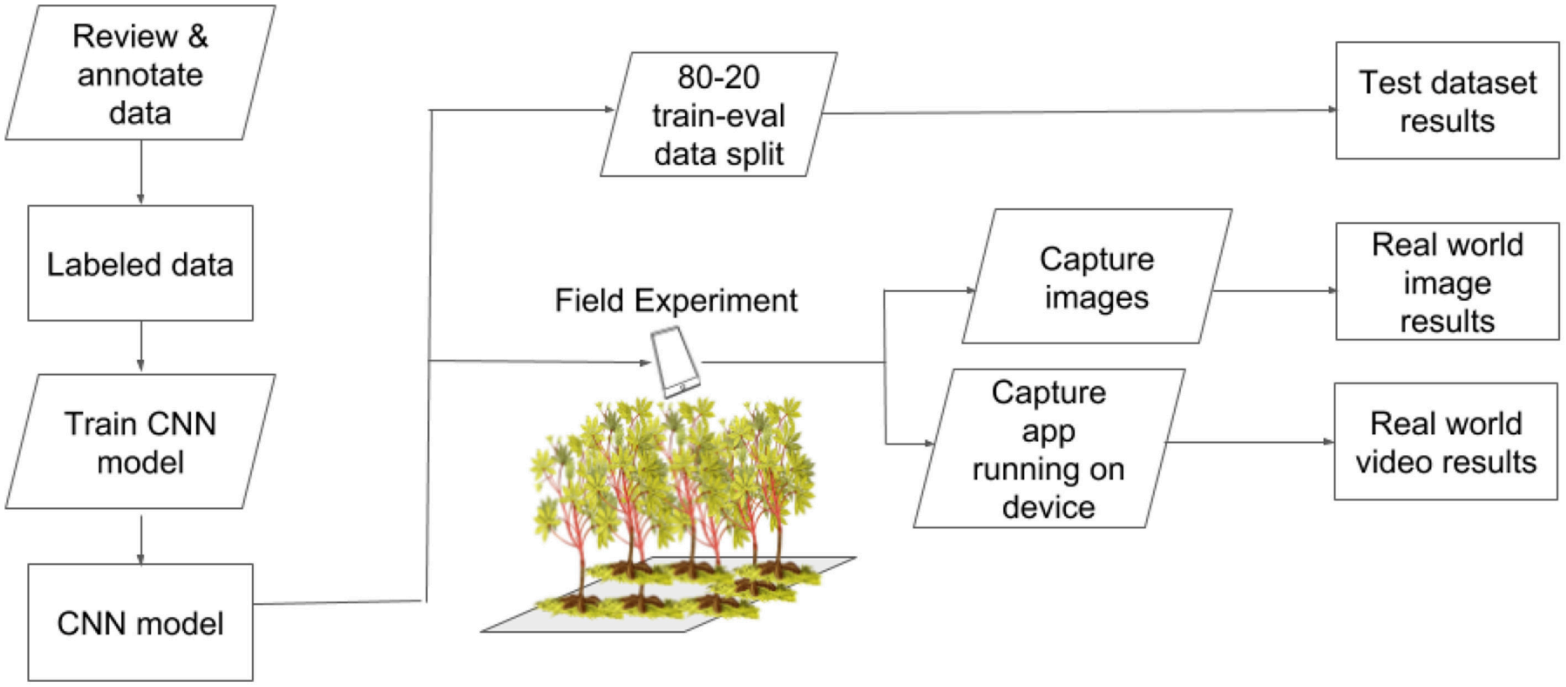
Experimental workflow to test performance of a CNN object detection model for real-time plant disease diagnosis.

**Figure 3:**
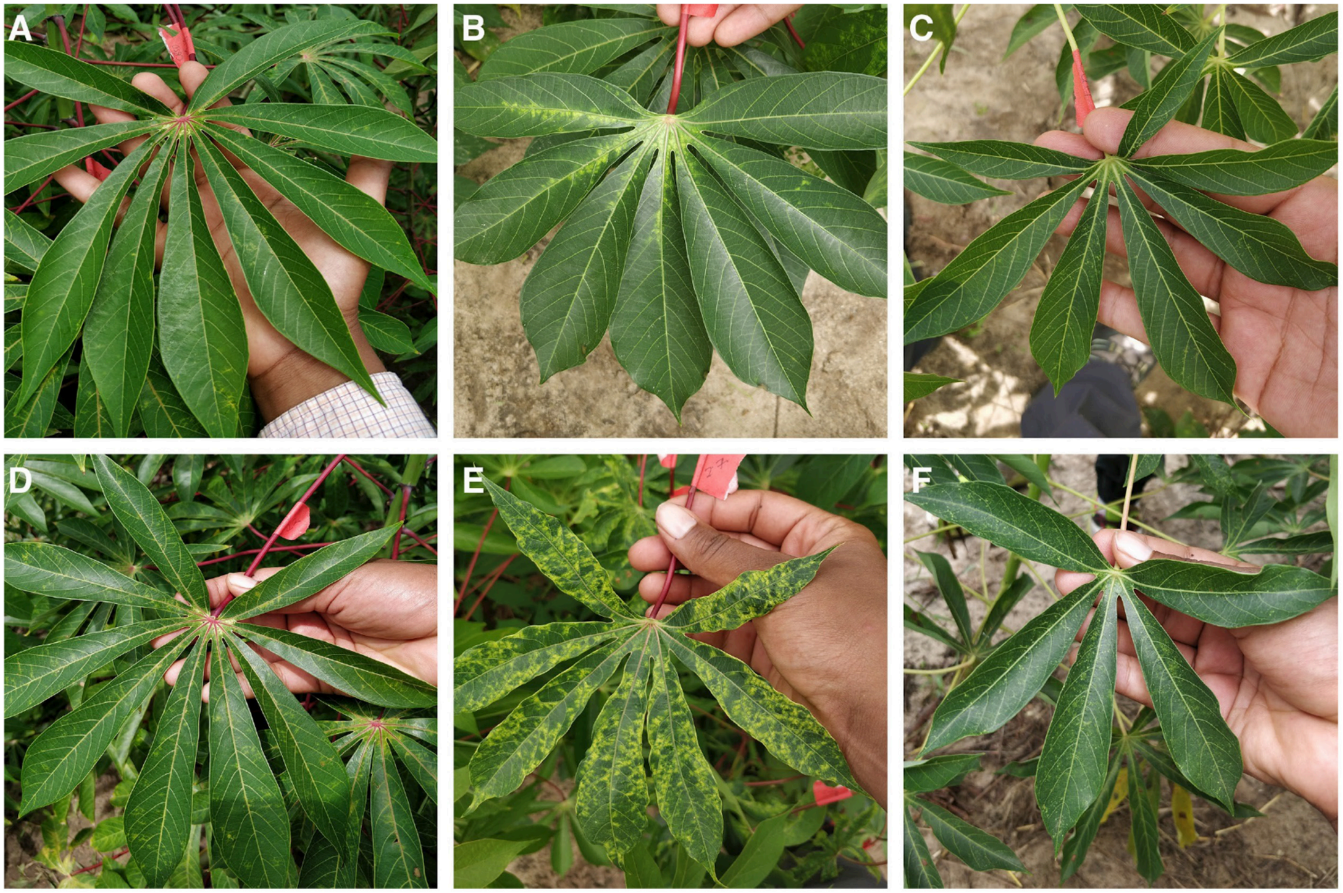
Examples images from field experiment showing mild symptoms **(A–C)** and pronounced symptoms **(D–F)** of CBSD, CMD, and CGM respectively.

We selected three classes for detection in the field - cassava mosaic disease (CMD), cassava brown streak disease (CBSD), and green mite damage (CGM) (Legg, 2012). For simplicity, CMD, CBSD and CGM are referred to collectively as disease in the subsequent text. These diseases were selected as they are the major constraints to cassava production in sub-Saharan Africa (Onzo et al., 2005; Legg et al., 2006). Within each disease class, an IITA cassava disease expert identified 40 validation leaves which were split into 20 mild symptom leaves and 20 pronounced symptom leaves. The work flow design of the experiment is shown in Figure 2. Examples of the leaves in this dataset are shown in Figure 3. The average number of leaflets per leaf was 6, resulting in an average of 120 objects per disease/severity group. Where possible, all leaves for each disease were flagged on the same variety of cassava. First, images of each of the 120 leaves were taken with a study experiment mobile device and model inference was run on a desktop to calculate performance metrics. Second, the mobile app was used for model inference in real time for the 120 leaves in overcast or cloudy conditions during the day. Model inference on the mobile app ranged from 50 to 200 ms. In order to evaluate the performance of the app, while the app was running on the mobile device, the device screen was recorded for 10 s using a free Android screen capture application. For simplicity, recordings of the app running on the mobile device are referred to from here on as “screen capture videos.” If weather conditions were sunny, an umbrella was used to shade leaves to obtain consistent light conditions across diseases. Leaves were also wiped to ensure surface was free of water and dirt. The mobile CNN model was deployed on a Samsung Galaxy S5 Android device using the Tensorflow Demo App. The phone was held parallel to the leaf, at a distance such that all leaflets were visible in the frame. A screen capture was recorded for the first 10 s where the model was shown the leaf and bounding boxes were proposed. These videos were then downloaded and used to calculate the real world video performance metrics for the model and this data are available upon request. For all experiments, detection boxes were displayed when model confidence was 85% or greater.

### 2.1. Data Preprocessing

The cassava leaf dataset of JPEG images were taken with a Sony Cybershot 20.2-MP digital camera. Complete details of this dataset were previously reported in Ramcharan et al. (2017). For this study, IITA cassava experts extracted 2,415 images from the dataset based on the visibility of the most severe symptoms of each class. This was done to test the potential for the model to learn from the clearest examples of the symptoms and then detect milder symptoms, which would be beneficial for more timely detection of diseases. The dataset totaled seven class labels as follows: three disease classes—cassava mosaic disease (CMD) (391 images), cassava brown streak disease (CBSD) (395 images), and brown leaf spot (BLS) (130 images), two mite damage classes—cassava green mite damage (GMD) (435 images), and red mite damage (RMD (351 images), and one nutritional deficiency class (NUTD) (336 images).

LabelImg (Tzutalin, 2015), an open source graphical annotation tool for manually drawing and labeling object bounding boxes in images, was employed to draw ground truth bounding boxes and create corresponding xml files with stored xmin, xmax, ymin, ymax data for each ground truth box. Images and corresponding xml files were then converted to TFRecord files to be implemented in the Tensorflow environment. TFRecord files combine all images and annotations into one file, thereby reducing training time as it eliminates the need to open individual files. Each of the three models were then trained to 500 epochs.

### 2.2. CNN Model

We evaluated the performance of the mobile CNN model built using standard precision metrics as well as a field-based independent evaluation on a mobile device. For the object detector model architecture, we selected the Single Shot Multibox (SSD) model with the MobileNet detector and classifier (Liu et al., 2016). This model was used as it is one of the fastest object detection models available through Tensorflow (Google, 2017). An SSD model performs the tasks of object localization and object classification in a single forward pass–a key component in providing real time object recognition on a mobile device (Mohanty et al., 2016). A pre-trained SSD model checkpoint trained on the COCO dataset (Common Objects in Context) was downloaded from Tensorflow's Detection Model Zoo (Google, 2017) and transfer learning was employed to fine tune the model parameters. COCO is a large scale object detection, segmentation and captioning dataset comprised 330 K images, 1.5 million object instances, and 80 object classes. Each model was trained up to 500 epochs using a batch size of 15 on 2 NVIDIA Tesla V100 GPU's in Azure, the Microsoft cloud computing and storage platform. An 80–20 training-evaluation data split was used as this data partitioning scheme produced the best results for cassava disease classification (Ramcharan et al., 2017). The SSD model parameters were selected as follows: initial learning rate of 0.004, weighted sigmoid classification loss function, and weighted smooth L1 localization loss function between the predicted bounding box (l) and ground truth bounding box (g). These loss functions are computed based on default bounding boxes-a set of boxes with specified aspect ratios. The classification loss function measures the model's confidence in classifying pixels within a default bounding box into one class (Liu et al., 2016). Localization loss measures the geometric distance between a default bounding box and the ground truth annotation bounding box. The overall loss function is a weighted combination of the classification loss (classif) and the localization loss (loc) with the weight for the localization loss, α,set to 1 (Liu et al., 2016) (Equation 1).

(1) $$\begin{array}{r} L\left(x,c,l,g\right)\;=\;\frac{1}{N}\left(L_{classif}f\left(x,c\right)+\alpha L_{loc}\left(x,l,g\right)\right) \end{array}$$

The maximum number of hard mining examples was set to 3,000, and the ratio between negative and positive examples was left at the default value of 3:1. A positive example is a proposed box with an annotated object of interest and correct box scale. A negative example is a proposed box with no annotated objects of interest and an incorrect box scale. The default box generator was applied to 6 different convolution layers with a minimum and maximum scale of 0.2 and 0.95 respectively. The default boxes were generated with fixed aspect ratios 1.0, 2.0, 0.5, 3.0, and 0.333. The complete details of the SSD model design principles are provided in Liu et al. (2016). In order to perform real time inference on a mobile device, images were resized to 300 × 300 pixels before being fed into the network.

## 3. Results

Validation results for the model are provided in three ways and results are presented for the three disease classes studied in the field. First, precision and recall results with an 80–20 training-testing data split are reported in Table 1. The mean average precision (mAP) is the average across N classes of the true positive class labels divided by the total number of objects labeled as belonging to the positive class (Equation 2). The mean average recall (mAR) is defined as the average across N classes of the number of true positive class labels divided by the total number of ground truth positive class labels (Equation 3). These metrics were calculated assuming the cost of a false positive was equal to no predictions for a leaflet. For the disease classes of interest, the CNN detection model achieves 94 ± 5.7% (mean±s.d.) mAP (67.6 ± 4.7% mAR) for the test dataset. Second, the results of the mAP and mAR evaluation using 120 images (comprising 742 leaflet “objects”) of the field experimental leaves run on a desktop are reported. For pronounced symptomatic leaves, the model achieves 91.9 ± 10% mAP and 39.3 ± 10.9% mAR, while for mild symptomatic leaves, the model achieves 75.0 ± 19% mAP and 16.8 ± 10.9% mAR. Third, the results of the precision and recall evaluations from 120 screen capture videos (of the 742 leaflet objects) are reported. For pronounced symptomatic leaves, the model achieves 89.6 ± 10% mAP and 39.8± 19.9% mAR, while for mild symptomatic leaves, the model achieves 81.2 ± 27% mAP and 15.8 ± 10.3% mAR. These results show that the model maintains its average precision for pronounced symptoms in real world images and video and there is a small drop in performance for mild symptoms. With respect to precision the mobile CNN model does slightly better on cassava mosaic disease (CMD) symptoms and slightly worse on cassava brown streak disease (CBSD) and cassava green mite damage (CGM). The model recall is reduced by almost half its test dataset value in real world images and video of pronounced symptoms. This reduction is almost four times as large in mild symptom real world images and video.

**Table 1:** Mean average precision of CNN model for real world images and video.

	**Severity**	**mAP**	**Class average precision**		
			**CBSD**	**CMD**	**CGM**
Test dataset	Pronounced	94 ± 5.7	87.5	98.3	96.2
Real world image	Pronounced	91.9 ± 10	81.8	97.9	89.1
Real world video	Pronounced	89.6 ± 10	81.0	100	94.7
Real world image	Mild	75.0 ± 19	61.1	100	82.6
Real world video	Mild	81.2 ± 27	45.7	100	79.3
		**mAR**	**Class average recall**		
			**CBSD**	**CMD**	**CGM**
Test dataset	Pronounced	67.6 ± 4.7	62.7	68.2	72.0
Real world image	Pronounced	39.3 ± 10.9	32.5	33.6	51.9
Real world video	Pronounced	39.8 ± 19.9	21.0	25.0	57.3
Real world image	Mild	16.8 ± 10.9	21.1	4.40	25.0
Real world video	Mild	15.8 ± 10.3	11.3	8.50	27.6

The F-1 scores (Equation 4) are also reported in Table 2. The F-1 score takes into account false positive and false negatives as it is a weighted average of precision and recall. The F-1 scores are 0.54 and 0.48 for pronounced symptoms in real world images and video, respectively. For mild symptoms the F-1 scores are 0.26 and 0.25 for real world images and video, respectively. Using the test dataset F-1 score as the baseline for comparison, the results show there is a 32% drop in F-1 score moving from the test dataset to pronounced symptoms in real world images (the closest data to the training data). For pronounced symptoms in real world video the F-1 score is reduced by 39%. Comparing the test dataset to real world mild symptoms the F-1 score drops by 67% for images and video. These results show there is a noticeable drop in mobile CNN model performance from the test dataset to real world conditions with real world images performed slightly better than real world video for both pronounced and mild symptoms.

(2) $$\begin{array}{rcl} mAP & = & \frac{1}{N}\overset{N}{\underset{n=1}{\sum }}\frac{Positive\;Detections}{All\;Positive\;Detections} \end{array}$$

(3) $$\begin{array}{rcl} mAR & = & \frac{1}{N}\overset{N}{\underset{n=1}{\sum }}\frac{True\;Positive\;Detections}{Ground\;Truth\;Positive\;Labels} \end{array}$$

(4) $$\begin{array}{rcl} F-1Score & = & \frac{2*Recall*Precision}{Recall+Precision} \end{array}$$

**Table 2:** F-1 Scores for real world evaluation.

**Test dataset**	**Severity**	**F-1 score**
Test dataset	Pronounced	0.79
Real world image	Pronounced	0.54
Real world video	Pronounced	0.48
Real world image	Mild	0.26
Real world video	Mild	0.25

We also calculated accuracies for the mobile CNN model on the real world images and screen capture videos. The accuracy is calculated as the percent of the examples the model correctly detects i.e., the proportion of the observations where the predicted and ground truth annotations match.

Images and videos were reviewed by a cassava disease expert in order to calculate these metrics. Accuracy results for the mobile CNN model run on 120 images (comprising 742 leaflet “objects”) of the field experimental leaves are reported in Table 3. For pronounced symptomatic leaves, the model achieves 80.6 ±4.10% accuracy, while for mild symptomatic leaves, the accuracy reduces to 43.2 ± 20.4%. The accuracy evaluation is then repeated using 120 screen capture videos of the mobile CNN model running real time in the field. For pronounced symptomatic leaves, the model achieves 70.4 ± 22.5% accuracy, while for mild symptomatic leaves, accuracy reduces to 29.4 ± 12.2%. Based on class average accuracy, the mobile CNN model does the best with CGM, followed by CMD, then CBSD.

**Table 3:** Accuracy results for the mobile CNN model for real world images and video.

	**Severity**	**Accuracy**	**Class average accuracy**		
			**CBSD**	**CMD**	**CGM**
Real world image	Pronounced	80.6 ± 4.10	76.1	83.9	81.7
Real world video	Pronounced	70.4 ± 22.5	45.9	90.3	74.0
Real world image	Mild	43.2 ± 20.4	61.1	21.1	47.5
Real world video	Mild	29.4 ± 12.2	23.9	20.8	43.4

**Figure 4:**
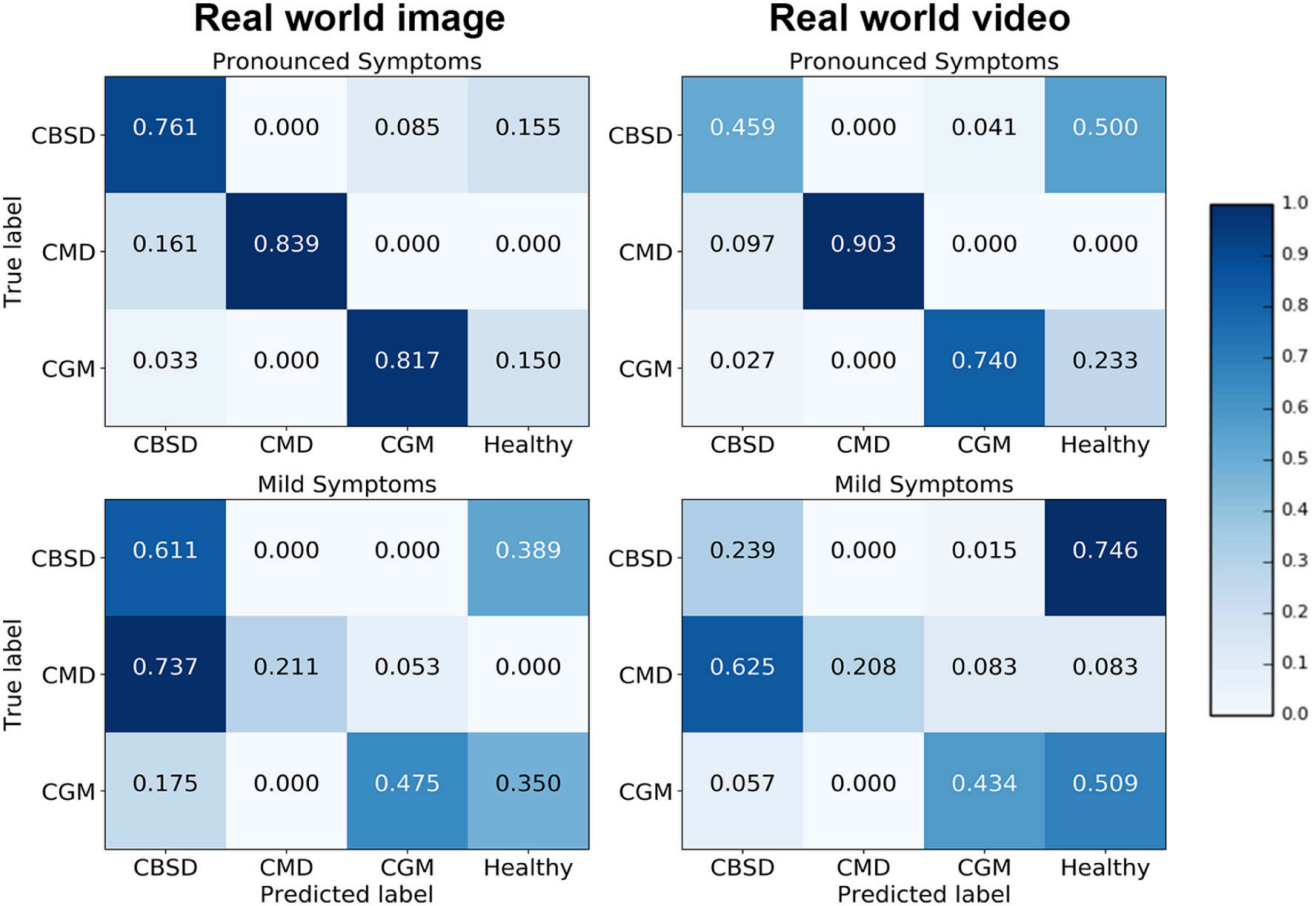
Confusion matrices for real world mobile images and real world mobile video.

Confusion matrices for the real world image and video model experiments give a more detailed analysis on how the model performance changes for different class/severity categories. In the confusion matrix plots in Figure 4, the rows correspond to the true class, and the column shows the model predicted class. The diagonal cells show the proportion (range 0–1) of the examples the model correctly detects i.e., the proportion of the observations where the predicted and ground truth annotations match. The off-diagonal cells show where the model made incorrect predictions.

The diagonal cells of the confusion matrices for leaves with pronounced symptoms show the model performs, as expected, much better where the symptoms are pronounced compared to leaves where the symptoms are mild. The biggest drop in accuracy going from pronounced symptoms to mild symptoms was for CMD for both image and video inferences of the model. This result can be due to the distortions of the leaf shape that occur for pronounced symptoms of CMD which are less obvious where symptoms are mild; the leaf distortion effect of disease does not occur for CBSD and only occurs in severe damage by CGM (not captured in this experiment). These results suggest that if symptoms change significantly during the different stages of infection, a model trained on one stage will be less reliable in detecting a different stage of infection of a disease. Comparing the performance of the model on images and video, the model accuracy is not significantly different for CMD and CGM classes but there is a surprising drop in accuracy for CBSD. There is a significant difference in CBSD accuracy for images (*M* = 0.73, *SD* = 0.10) and video (*M* = 0.35, *SD* = 0.11); *t*_(36)_ = 6.64, *p* = 0.0. This may be due to the subtlety of infection of CBSD. CBSD symptoms are not localized on an area of the leaf (unlike CMD and CGM). The contrast in color and patterns of CBSD symptoms is less pronounced than CMD and CGM. This could make CBSD symptoms more sensitive to motion blur and compression artifacts causing frame-to-frame variability, even though videos appear smooth to the eye (Tripathi et al., 2016). The accuracy of the model could be improved with domain adaptation algorithms (Ben-David et al., 2010) to reduce this performance gap. The confusion matrices also show that the mobile CNN model confuses mild symptoms with healthy leaves for symptoms that do not result in distortions in leaf shape. For CMD, the model was very effective in detecting severe symptoms, but very poor with mild symptoms. The leaf distortion that occurs in severe CMD infection makes identification of that class straightforward, but where symptoms are mild and leaf distortion was absent, there was a high level of confusion with CBSD (false positive rate is high for CBSD). This is unsurprising, as the difficulty of distinguishing between mild symptoms of these two diseases is a common problem faced in real-world field situations by cassava researchers.

## 4. Discussion and Conclusion

In this study we evaluate the performance of a CNN model deployed offline in real time on a mobile device to detect foliar symptoms of cassava pests and diseases. Using the single-shot detector model, a CNN architecture optimized for mobile devices, we assess the performance of the model to detect pronounced and mild symptoms of 3 disease classes. In both severities we see a decrease in F-1 score comparing the test dataset results to real world images and video. The decrease in performance was mostly due to the decrease in recall as the models overall maintained precision in real world conditions. Accuracy results also reflected the decrease in performance moving from real world images to video. The performance of the model in the mobile video changed depending on the difficulty of the visual diagnostic task. In order to obtain higher accuracy detections, there are a number of potential solutions-feed the mobile CNN model images collected on a mobile device instead of real time video assessment, train on video images saved directly from the mobile app, or employ domain adaptation algorithms to improve performance. The mobile CNN model also decreased in performance for mild symptoms, with accuracies decreasing more for diseases that change the leaf characteristics considerably during different stages of infection. In order to create a model that can detect mild symptoms of disease, images of mild infection are needed for model training. Collecting these images based solely on visual characteristics may be difficult as some groups of image classes e.g., viral diseases, may look similar to each other where infection is not severe. PCR tests may be needed to complement images to confirm symptoms. This study demonstrates the need to evaluate mobile CNN performance in realistic plant disease diagnostic operating conditions, with multiple performance metrics in order to validate models with data encountered in typical settings. Based on our results, we also recommend mobile CNN models to be used on the specific type of data with which it was trained until sufficient training examples exist from diverse data sources to better capture the diversity of data that occur in the real world.

## Author Contributions

AR conceptualized experiment, selected algorithm, collected and analyzed data, and wrote manuscript. PM trained algorithms, collected and analyzed data, and wrote manuscript. KB analyzed data. NM and MN collected data. LM, JL, and DH are project supervisors. We are grateful to Pete Warden of the Google Tensorflow mobile team for technical insight.

### Conflict of Interest Statement

The authors declare that the research was conducted in the absence of any commercial or financial relationships that could be construed as a potential conflict of interest.
