# Supplementary material for: A Mobile-Based Deep Learning Model for Cassava Disease Diagnosis
Source: Front Plant Sci. 2019 Mar 20;10:272. doi: 10.3389/fpls.2019.00272 (PMC6436463; doi:10.3389/fpls.2019.00272)
Supplement: Supplementary file 1 [file Data_Sheet_1.PDF]

Supplementary Material: Article Title

Amanda Ramcharan*, Peter McCloskey, Kelsee Baranowski, Neema Mbilinyi, Latifa Mrisho, Mathias Ndalahwa, James Legg, and David Hughes

*Correspondence:
Author Name: Amanda Ramcharan
amr418@psu.edu, dph14@psu.edu

David Hughes: dph14@psu.edu

1 ANNOTATION EXPERIMENT RESULTS

The mean average precision (mAP) across all classes as well as for each class for the annotation experiment are reported in Supplmentary Table 1. These metrics were then repeated for the in-field model assessment of the mobile CNN model for annotation style 1 (whole leaflet).

2 SUPPLEMENTARY TABLES AND FIGURES

Table S1. Mean average precision for 3 annotation styles studied

Annotation Style	mAP	Class average precision		
		CBSD	CMD	CGM
1 - whole leaflet	71.9	66.4	66.0	83.2
2 - within leaflet	13.6	17.1	11.9	11.8
3 - combined whole + within leaflet	10.9	15.0	9.33	8.27
